# Effects of apatite particle size in two apatite/collagen composites on the osteogenic differentiation profile of osteoblastic cells

**DOI:** 10.3892/ijmm.2013.1516

**Published:** 2013-10-02

**Authors:** WATARU HATAKEYAMA, MASAYUKI TAIRA, NAOYUKI CHOSA, HIDEMICHI KIHARA, AKIRA ISHISAKI, HISATOMO KONDO

**Affiliations:** 1 Department of Prosthodontics and Oral Implantology, Iwate Medical University School of Dentistry, Morioka, Iwate 020-8505, Japan; 2 Department of Biomedical Engineering, Iwate Medical University, Yahaba, Shiwa-gun, Iwate 028-3694, Japan; 3 Division of Cellular Biosignal Sciences, Department of Biochemistry, Iwate Medical University, Yahaba, Shiwa-gun, Iwate 028-3694, Japan

**Keywords:** SaOS-2 osteoblastic cells, apatite size, collagen composite, osteogenic differentiation, gene expression

## Abstract

The development of new osteoconductive bone substitute materials is expected in medicine. In this study, we attempted to produce new hydroxylapatite (HAP)/collagen (Col) composites using two HAP particles of different sizes and porcine type I collagen. The two HAP particles were either nano-sized (40 nm in average diameter; n-HAP) or had macro-pore sizes of 0.5–1.0 mm in length with fully interconnected pores (m-HAP). The aim of this study was to investigate the effects of apatite particle size in two HAP/Col composites on the osteogenic differentiation profile in osteoblast-like cells (SaOS-2). We created a collagen control sponge (Col) and two HAP/Col composite sponges (n-HAP/Col and m-HAP/Col) using freeze-drying and dehydrothermal cross-linking techniques, and then punched out samples of 6 mm in diameter and 1 mm in height. The SaOS-2 cells were cultured on three test materials for 1, 2, 3 and 4 weeks. Total RNA was extracted from the cultured cells and the expression of osteogenic differentiation-related genes was evaluated by reverse transcription PCR (RT-PCR) using primer sets of alkaline phosphatase (ALP), type 1 collagen (COL1), bone sialoprotein (BSP) and osteocalcin precursor [bone gamma-carboxyglutamate (gla) protein (BGLAP)] genes, as well as the β-actin gene. The cells were also cultured on Col, n-HAP/Col and m-HAP/Col specimens for 1 and 4 weeks, and were then observed under a scanning electron microscope (SEM). The experimental results were as follows: RT-PCR indicated that osteogenic differentiation, particularly the gene expression of BSP, was most accelerated when the cells were cultured on n-HAP/Col specimens, followed by m-HAP/Col, whilst the weakest accelaeration was observed when the cells were cultured on Col specimens. As shown by the SEM images, the SaOS-2 cells were fibroblastic when cultured on Col specimens for up to 4 weeks; they were fibroblastic when cultured on n-HAP/Col specimens for 1 week, but appeared as spheroids, while actively phagocytizing n-HAP particles at 4 weeks; however, they appeared as deformed fibroblasts when cultured on m-HAP/Col specimens, detached from the particles. Despite limited experimental results, our study suggests that n-HAP/Col may be employed as a new osteoconductive bone substitute material.

## Introduction

Hydroxylapatite (HAP)/collagen (Col) composites have often been utilized as bone substitute materials in dentistry and orthopedic surgery for the regeneration of damaged hard tissue (1). HAP has the capability of excellent osteoconduction and slow biodegradation (2), while Col is the bio-absorbable scaffold used to bind HAP (3). Different sizes of HAP can be used in HAP/Col composites (4,5).

Nano-HAP (n-HAP) particles have attracted attention in the field of biomaterials, as they are considered to have unknown advantageous capabilities (4,6). It is, however, necessary to mix n-HAP with Col to form a bone substitute material (6). Macro-pore sized HAP (m-HAP) is now solely employed as particulate bone substitute material (5,7), and can also be mixed with Col for new biomaterial. However, few systematic studies have evaluated the osteoconductive capability of n-HAP/Col and m-HAP/Col composites (4).

To achieve osteoregeneration following the implantation of HAP/Col composites in the bone defect area, it is required that osteoblasts adhere to and differentiate into a final matured stage in contact with HAP/Col composites (8). It is known that osteoblasts differentiate following a characteristic step-wise sequence, while secreting respective phenotype marker enzymes/proteins in multi-lineage differentiation [i.e., early-stage alkaline phosphatase (ALP) and type 1 collagen (COL1); and middle-to-late-stage bone sialoprotein (BSP) and the osteocalcin precursor, bone gamma-carboxyglutamate (gla) protein (BGLAP)] (9). Reverse transcription PCR (RT-PCR) is a sophisticated technique which can be used to evaluate the expression of genes (mRNAs) which produce marker enzyme/proteins in osteoblasts cultured on implant materials (10–12).

The aim of the current study was, therefore, to evaluate the expression of four selected osteogenic differentiation-related genes (i.e., ALP, COL1, BSP and BGLAP) in osteoblast-like cells (SaOS-2) cultured on Col, n-HAP/Col and m-HAP/Col composites, using a world class standard RT-PCR machine in order to determine the effects of two HAP/Col composites containing different HAP particle sizes (i.e., n-HAP/Col and m-HAP/Col) on the osteogenic differentiation of osteoblasts. Furthermore, SaOS-2 cells cultured on three test materials were observed under a scanning electron microscope (SEM) in order to osberved the cellular morphological changes that occurred during the osteogenic differentiation of the SaOS-2 cells.

## Materials and methods

### HAP particles

The n-HAP particles used were mono-dispersed HAP ceramics with an average diameter of 40 nm (MHS-00405 type nano-SHAp; SofSera Corp., Tokyo, Japan), as previously described (13). The m-HAP particles used were novel interconnected porous HAP ceramics with a large dimension of 0.5–1 mm (DK03-001, IP-CHA; Covalent Materials Co., Tokyo, Japan), as previously described (5). Both HAP particles were of high purity, fired at >800°C and highly crystalline, as previously described (5,13). The basic intrinsic difference between the n-HAP and m-HAP particles lied in their size.

### Preparation of HAP/Col composites

Collagen pellets produced from porcine skin (NMP Collagen PS; Nippon Meat Packers Inc., Tokyo, Japan) (1 g) were dissolved in distilled water (28 ml) in a 50 ml polystyrene conical tube at 4°C. The formed acidic solution was neutralized by 0.1 N NaOH solution (6.5 ml) in a disposable rectangular plastic plate (100×70×12 mm) in order to obtain a collagen gel of pH 7.5. The n-HAP or m-HAP powder (1.5 g) was then manually mixed with collagen gel using a plastic spatula. The HAP/Col composite gel was then frozen at −80°C for 3 h, and freeze-dried using a freeze-drier (FD-5N; Eyela, Tokyo, Japan) for 12 h. The formed composite sponge stored in a holed stainless steel case was subsequently cross-linked by dehydrothermal treatment at 140°C for 24 h in a vacuum drying oven (VO-300; Asone, Osaka, Japan). From the composite sponge sheet, discs 6 mm in diameter and 1 mm thickness were punched out with a hole puncher. Discs stored in exclusive pouches were sterilized by ethylene oxide gas and kept in a vacuumed desiccator before the cell culture experiments.

### Culture of SaOS-2 cells on HAP/Col composites

Human osteoblast-like cells (SaOS-2) (RCB0428; Riken BioResource Center Cell Bank, Tsukuba, Japan) were regularly cultured in Eagle’s α-modified minimum essential medium (Invitrogen, Carlsbad, CA, USA) supplemented with 10% fetal bovine serum (Cat. no. 10099-141; Invitrogen) and 2% antibiotics (penicillin-streptomycin-amphotericin; Cat. no. 5240-096; Invitrogen) in a 5% CO_2_ incubator at 37°C. After subconfluence, the cells were collected by trypsinization with phosphate-buffered saline [PBS(−)] containing 0.08% trypsin (Cat. no. 15090-046; Invitrogen) and 0.14% EDTA (Cat. no. 15576-010; Invitrogen) and subcultured at 1:3 ratios.

The culture of SaOS-2 cells on the Col, n-HAP/Col and m-HAP/Col specimens was carried out as follows: The SaOS-2 cells (1×10^4^) in 200 μl medium were inoculated on three test materials held in wells of a 96-well polystyrene culture dish. The SaOS-2 cells on the test materials were then continuously cultured for 1, 2, 3 and 4 weeks in a 5% CO_2_ incubator at 37°C, while the medium was changed twice each week.

### Selection of osteogenic differentiation-related genes

As osteogenic differentiation-related phenotype markers, five genes were selected, including the β-actin gene, which was used as the control. The ALP and COL1 genes, and the BSP and BGLAP genes were used as early-stage and middle-to-late stage osteogenic differentiation phenotype markers, respectively (11). The formal nomenclatures and GenBank accession numbers (in the genetic sequence database, National Institutes of Health, Bethesda, MD, USA) (NM_) of the five genes were as follows: β-actin, *Homo sapiens* actin, beta (ACTB), mRNA, NM_001101.3; ALP, *Homo sapiens* alkaline phosphatase, liver/bone/kidney, transcript variant 1, mRNA, NM_000478.3; COL1, *Homo sapiens* collagen, type I, α1 mRNA, NM_000088.3; BSP, *Homo sapiens* integrin-binding sialoprotein, mRNA, NM_004967.3; and BGLAP, bone gamma-carboxyglutamate (Gla) protein (osteocalcin precursor), NM_199173.

### RT-PCR

Total RNA was extracted from the cells using ISOGEN reagent (Nippon Gene, Tokyo, Japan). Reverse transcription was performed using the PrimeScript RT reagent kit (Takara Bio., Inc., Ohtsu, Japan). The mRNA expression levels of the five genes were determined by RT-PCR using SYBR Premix Ex *Taq* II (Takara Bio, Inc.) and the Thermal Cycler Dice Real Time System TP8000 (Takara Bio, Inc.) and five primers (Table I) designed by the Perfect Real Time support system (Takara Bio, Inc.). The primer set for BGLAP was specifically created upon our request, while those for the other four genes were ready-made in the support system (Takara Bio, Inc.). For each test run, cDNA derived from 50 ng total RNA was used. After an initial denaturation at 95°C for 30 sec, a two-step cycle procedure was used (denaturation at 95°C for 5 sec, annealing and extension at 60°C for 30 sec) for 40 cycles. Gene expression levels were normalized according to the expression level of the β-actin gene. Relative amounts (RQ values) of each mRNA in each sample were calculated using the ΔΔCt method. The gene expression analyses were duplicated. To ensure reproducibility, each sample was analyzed in triplicate. Data are presented as the means ± standard deviation.

### Statistical analysis

Statistical analysis was carried out using the Student’s t-test and a value of α=0.05 was considered to indicate a statistically significant difference.

### Observations of SaOS-2 cells cultured on HAP/Col composites with SEM

SaOS-2 cells cultured on Col, n-HAP/Col and m-HAP/Col specimens for 1 and 4 weeks were freeze-dried at room temperature for 1 h, following fixation in 2.5% glutaraldehyde solution, fixation in 1% osmium solution, dehydration in graded alcohols, infiltration by t-butyl alcohol and freezing at 0°C for 12 h. The cells cultured on the three test materials were then observed under an SEM (S-2300; Hitachi, Ibaragi, Japan).

## Results

### RT-PCR

#### Early-stage osteogenic differentiation marker genes (ALP and COL1)

**Figure 1:**
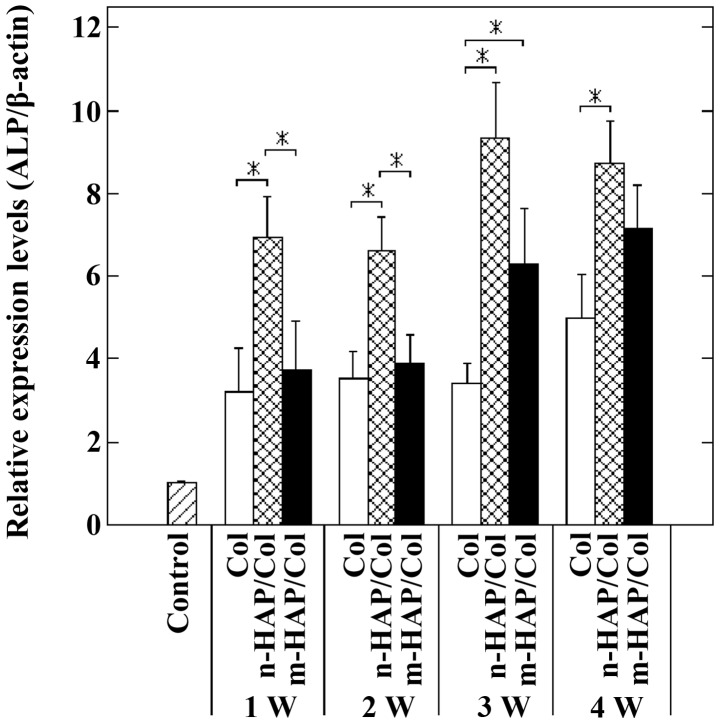
Expression of the alkaline phosphatase (ALP) gene in osteoblast-like cells (SaOS-2) cells cultured on collagen (Col), nano-sized hydroxylapatite (nHAP)/Col and macro-pore sized hydroxylapatite (m-HAP)/Col composites for 1, 2, 3 and 4 weeks (^*^p<0.05). The expression levels of the ALP gene in the SaOS-2 cells cultured on Col were greater than those of the control, but remained quasi-constant for up to 4 weeks. At 1 and 2 weeks, culture on n-HAP/Col markedly upregulated the expression of the ALP gene in the SaOS-2 cells compared with culture on Col and m-HAP/Col (p<0.05), and the expression levels of the ALP gene in the cells cultured on m-HAP/Col and Col were similarly lower. Thus, n-HAP/Col solely had the ability to rapidly induce early-stage osteogenic differentiation characterized by the expression of the ALP gene. At 3 and 4 weeks, the expression of the ALP gene in the cells cultured on n-HAP/Col tended to still be higher, followed by m-HAP/Col, while cells cultured on Col tended to have the weakest expression of ALP. These results demonstrate that n-HAP/Col has the ability to induce the early-stage osteogenic differentiation of SaOS-2 cells at 3 and 4 weeks, whilst the osteogenic differentiation of cells cultured on m-HAP/Col virtually only began at 3 weeks. W, week.

The expression of the ALP gene clearly distinguished the early-stage osteogenic differentiation of the SaOS-2 cells cultured on the three test materials. The n-HAP particles in the n-HAP/Col composite rapidly (from 1 week) and continuously (over a period of 4 weeks) accelerated early-stage osteogenic differentiation characterized by the expression of the ALP gene, compared with the m-HAP particles in the m-HAP/Col composite and Col (Fig. 1).

**Figure 2:**
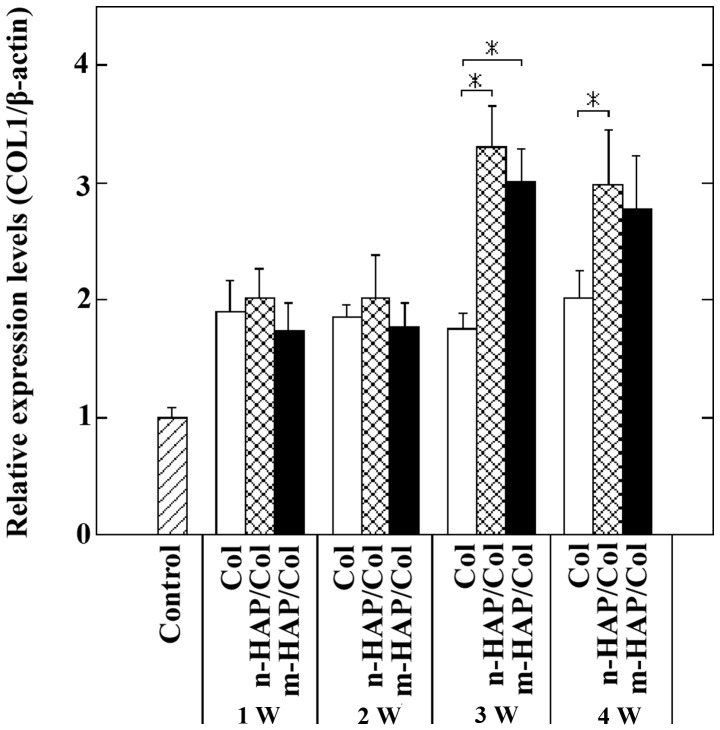
Expression of the type 1 collagen (COL1) gene in osteoblast-like cells (SaOS-2) cells cultured on collagen (Col), nano-sized hydroxylapatite (nHAP)/Col and macro-pore sized hydroxylapatite (m-HAP)/Col composites for 1, 2, 3 and 4 weeks (^*^p<0.05). The expression levels of the COL1 gene in cells cultured on Col were greater than those of the control, but remained quasi-constant for up to 4 weeks. At 1 and 2 weeks, the relative expression levels of the COL1 gene in SaOS-2 cells cultured on Col, n-HAP/Col and m-HAP/Col composites were all analogous to each other (approximately 1.9). It can be pointed out that at 1 and 2 weeks, both n-HAP/Col and m-HAP/Col composites had little effect on the upregulation of another early stage osteogenic differentiation marker, the COL1 gene. However, at 3 and 4 weeks, the expression levels of the COL1 gene in cells cultured on n-HAP/Col were significantly higher than those of cells cultured on Col (p<0.05), while those of cells cultured on m-HAP/Col tended to exceed those of cells cultured on Col. These results indicated that both n-HAP/Col and m-HAP/Col accelerated early-stage osteogenic differentiation characterized by the expression of the COL1 gene at 3 and 4 weeks. W, week.

On the other hand, at 1 and 2 weeks, the expression of the COL1 gene did not clearly display the difference in the osteogenic differentiation of the cells cultured on the three test materials. At 3 and 4 weeks, however, the SaOS-2 cells cultured on the n-HAP/Col and m-HAP/Col specimens were larger than those cultured on Col specimens with similar magnitude (Fig. 2).

#### Middle-to-late stage osteogenic differentiation marker (BSP and BGLAP) genes

**Figure 3:**
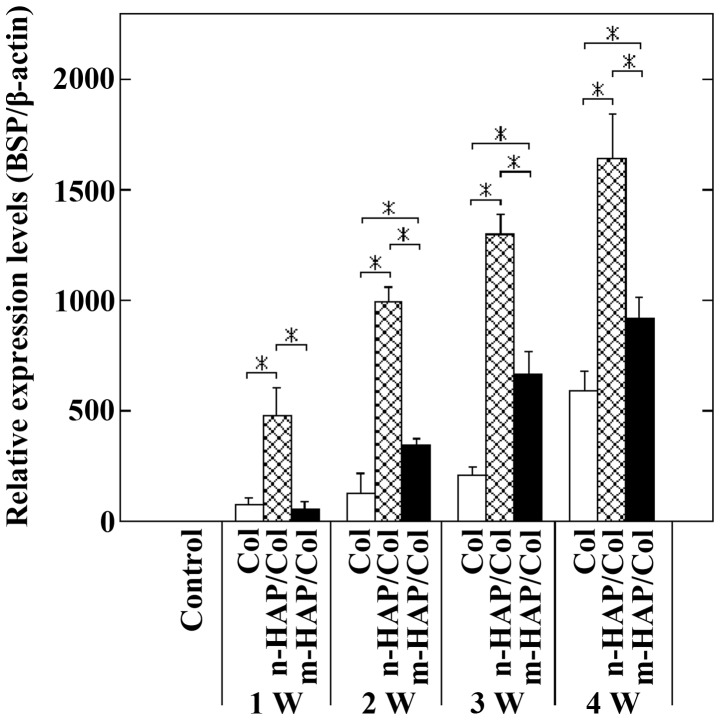
Expression of the bone sialoprotein (BSP) gene in osteoblast-like cells (SaOS-2) cells cultured on collagen (Col), nano-sized hydroxylapatite (nHAP)/Col and macro-pore sized hydroxylapatite (m-HAP)/Col composites for 1, 2, 3 and 4 weeks (^*^p<0.05). The expression levels of the BSP gene in the SaOS-2 cells cultured on Col were greater than those of the control, and gradually increased with incrementing the culture period up to 4 weeks. The expression levels of the BSP gene in the cells cultured on n-HAP/Col and m-HAP/Col also increased. It should be noted that the expression of the BSP gene in the cells cultured on n-HAP/Col was predominantly the greatest, followed by that in cells cultured on m-HAP/Col, while the cells cultuerd on Col had the weakest expression. It was demonstrated that n-HAP particles in the n-HAP/Col composite had the ability to accelerate middle-to-late stage osteogenic differentiation characterized by the expression of the BSP gene. It was confirmed that m-HAP/Col had limited capability to accelerate middle-to-late stage osteogenic differentiation, compared with n-HAP/Col. W, week.

The expression of the BSP gene was the most sensitive indicator of osteogenic differentiation of SaOS-2 cells cultured on the three test samples. The n-HAP particles in the n-HAP/Col composite significantly accelerated middle-to-late stage osteogenic differentiation, characterized by the expression of the BSP gene; this by far exceeded the osteogenic differentiation induced by culture on m-HAP/Col and Col specimens (p<0.05) (Fig. 3). It was observed that the m-HAP particles in the m-HAP/Col composite also had the capability to accelerate the said differentiation; however, the levels were decreased by >2-fold compared with those induced by n-HAP/Col.

**Figure 4:**
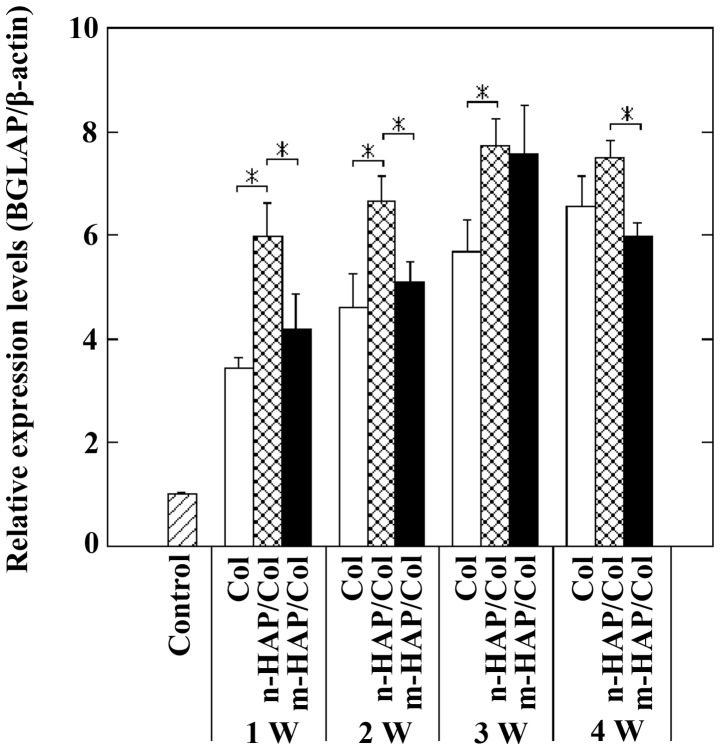
Expression of the bone gamma-carboxyglutamate (gla) protein (BGLAP) gene (osteocalcin precursor) in osteoblast-like cells (SaOS-2) cells cultured on collagen (Col), nano-sized hydroxylapatite (nHAP)/Col and macro-pore sized hydroxylapatite (m-HAP)/Col composites for 1, 2, 3 and 4 weeks (^*^p<0.05). The expression levels of the BGLAP gene in SaOS-2 cells cultured on Col were greater than those of the control, and gradually increased for up to 4 weeks. The expression of the BGLAP gene in the cells cultured on n-HAP/Col and m-HAP/Col slightly increased with time for up to 3 weeks, but slightly decreased at week 3 to 4. At 1 and 2 weeks, culture on n-HAP/Col markedly upregulated the expression of the BGLAP gene in SaOS-2 cells, compared with culture on Col and m-HAP/Col (p<0.05). It can be stated that n-HAP/Col solely had the ability to accelerate late-stage osteogenic differentiation from the beginning (at 1 week). At 3 and 4 weeks, the expression of the BGLAP gene in cells cultured on Col, n-HAP/Col and m-HAP/Col increased, but it became difficult to rank the ability to induce late-stage osteogenic differentiation among the three test samples. W, week.

At 1 and 2 weeks, the expression levels of the BGLAP gene in the SaOS-2 cells cultured on n-HAP/Col specimens were higher than those of cells cultured on m-HAP/Col and Col specimens (p<0.05), indicating that the n-HAP particles in the n-HAP/Col composites had the ability to rapidly induce late-stage osteogenic differentiation. At 3 and 4 weeks, however, the expression levels of the BGLAP gene in the cells cultured on the three test materials did not differ significantly, ranging between values of 6 to 7.5 (Fig. 4).

### SEM observations

#### Col

**Figure 5:**
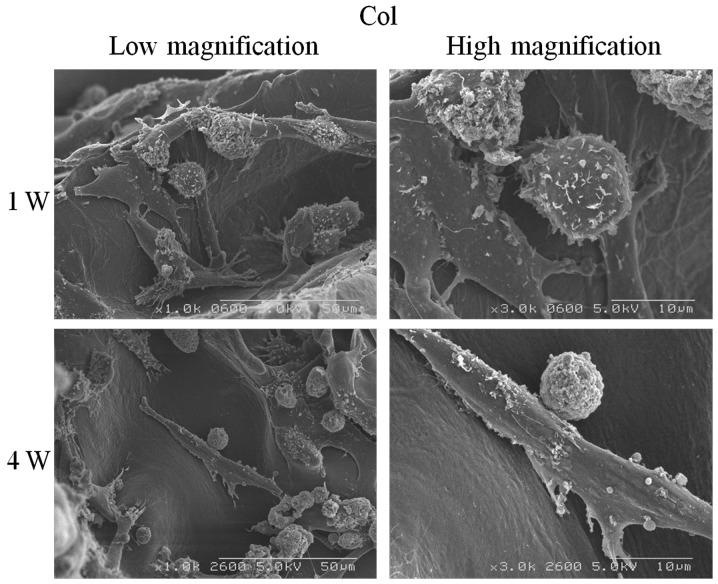
SEM images of osteoblast-like cells (SaOS-2) cells cultured on collagen (Col) composites for 1 and 4 weeks. At 1 week, the cells well adhered to and spread on the surfaces of Col, while extending the cellular process. At 4 weeks, the cells covered the surfaces of Col, occupying an area similar to that at 1 week, while losing the cellular process. Round particulates co-existed. The cells cultured on Col were fibroblastic for up to 4 weeks. W, week.

Col was a scaffold, on which the osteoblasts adhered and spread as fibroblasts. At 1 week, the SaOS-2 cells well covered the Col surfaces. At 4 weeks, the cells remained as fibroblasts with spherical particles (Fig. 5).

#### n-HAP/Col

**Figure 6:**
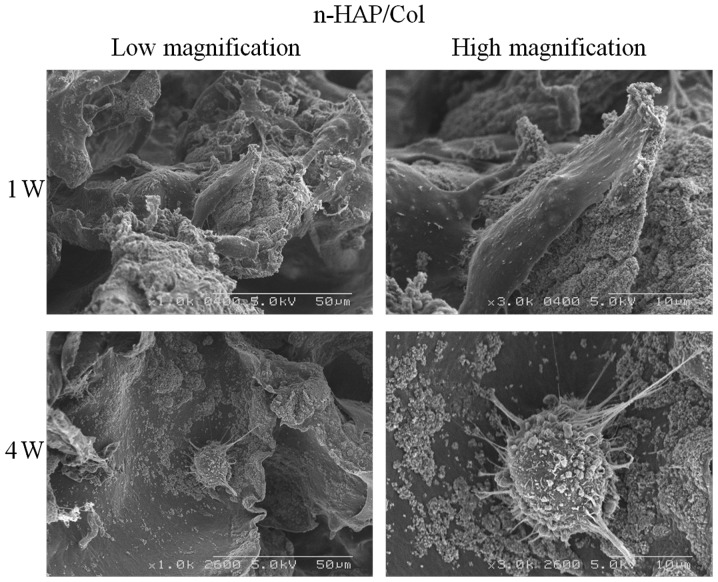
SEM images of osteoblast-like cells (SaOS-2) cells cultured on nano-sized hydroxylapatite (nHAP)/collagen (Col) composites for 1 and 4 weeks. At 1 week, the cells well adhered to and spread on the surfaces of agglomerated n-HAP particles, and appeared as fibroblasts. At 4 weeks, the cells actively phagocytized the n-HAP particles, while extending many cellular projections, and appeared as spheroids. W, week.

n-HAP caused the SaOS-2 cells to significantly change their shape as time progressed. At 1 week, the cells preferentially covered and spread over the agglomerated n-HAP particles in the n-HAP/Col composite as fibroblasts. At 4 weeks, however, the cells seemed to move widely and appeared as spheroids which actively phagocytized the n-HAP particles in the composite, while extending many projections (Fig. 6). This positively affected the osteogenic differentiation of the cells.

#### m-HAP/Col

**Figure 7:**
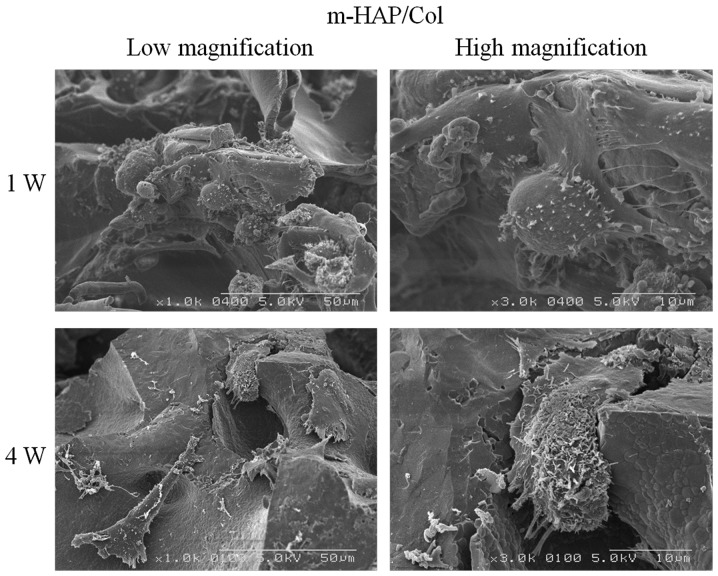
SEM images of osteoblasts-like cells (SaOS-2) cells cultured on macro-pore sized hydroxylapatite (m-HAP)/collagen (Col) composites for 1 and 4 weeks. At 1 week, the cells adhered to and had limited contact with the surfaces of m-HAP particle while maintaining the fibroblastic shape. At 4 weeks, the cells were isolated and appeared as deformed fibroblasts that resembled a sea cucumber. W, week.

Following culture for 1 week on the m-HAP/Col composite containing the m-HAP particles, it was relatively difficult for the fibroblastic SaOS-2 cells to adhere to the sponge and spread over. At 4 weeks, the cells appeared as deformed fibroblasts, detached from the m-HAP particles (Fig. 7).

## Discussion

SaOS-2 cells are used as a standard cell model of osteoblasts (14). We have already reported that the osteogenic differentiation of SaOS-2 cells was more accelerated following culture on an apatite-coated titanium plate surface than on a titanium plate, indicated by gene expression analyses using SaOS-2 cells (15). As SaOS-2 cells are originally found during early-stage osteogenic differentiation (i.e., ALP-positive cells), it was considered easy to evaluate the effects of materials and chemicals on the osteogenic differentiation of SaOS-2 cells at later stages.

It became clear from the results obtained that as marker genes, the ALP (Fig. 1) and BSP (Fig. 3) genes were excellent indicators of osteogenic differentiation, while the COL1 (Fig. 2) and BGLAP (Fig. 4) genes were ambiguous. It was emphasized that the BSP gene was the most outstanding indicator of the (middle-to-late stage) osteogenic differentiation of SaOS-2 cells cultured on HAP/Col composites (Fig. 3). We also examined the expression levels of a middle-stage marker, the opteopontin (SPP1) gene, in the cells cultured on three test materials, but were unable to determine the systematic effects of the materials on osteogenic differentiation. The reason for these phenomena is unclear, and is still under evaluation.

It should also be noted that higher levels of differentiation of the SaOS-2 cells were observed when the cells were cultured on three-dimensional Col compared with those cultured (control) on a two-dimensional polystyrene culture dish (Figs. 1–4). It appeared that Col provided three-dimensional space, allowing the SaOS-2 cells to have more intercellular connections, and also benefitting the acceleration of osteogenic differentiation (16).

The experimental results demonstrated that culture on n-HAP/Col composites significantly accelerated the osteogenic differentiation of SaOS-2 cells, followed by culture on m-HAP/Col composites; the lowest acceleration rate was observed with culture on Col composites. We considered that the reasons underlying these phenomena are the following:

n-HAP particles in the n-HAP/Col composite existed in an agglomerated state with a size of approximately 50 μm and were loosely bound (Fig. 6). It was observed that the SaOS-2 cells cultured for 1 week well adhered to and spread over the agglomerated n-HAP particles as fibroblasts (Fig. 6). It has been reported that several proteins, including cellular adhesion proteins (e.g., fibronectin) are abundantly and preferentially absorbed on n-HAP particles (17). These proteins may facilitate good adhesion and the movement of SaOS-2 cells. It was also observed that the SaOS-2 cells cultured for 4 weeks were isolated and became spheroids. These cells may have moved and actively phagocytized the n-HAP particles, while extending many projections (Fig. 6). Similar phagocytosis activity of osteoblasts has been reported by other studies (18). It can be hypothesized that the intracellular digestion of n-HAP particles may accelerate the osteogenic differentiation of SaOS-2 cells (4,18), partly due to the supply of calcium and phosphate ions (19), and the activation of cell signaling pathways (e.g., the MAPK pathway) (20). However, it should be noted that the digestion of n-HAP particles may cause the apoptosis of cells and the secretion of inflammatory cytokines by cells due to the production of reactive oxide species (ROS) (21,22). It may be necessary to balance the positive and negative effects of n-HAP particles by adjusting the quantities of n-HAP particles in n-HAP/Col composites. The use of the most well-balanced n-HAP/Col composite may lead to the development of a novel osteoconductive bone substitute material with rapid effects, which may significantly contribute to the treatment of patients with damaged or lost hard tissue in dental and orthopedic surgeries. Future uses of n-HAP particles in medicine may include the use of agents to release anti-microbial chemicals (23), DNA-transfection vectors (24) and biomaterial to induce angiogenesis at sites of ischemia (25).

m-HAP particles have gained a good reputation as particulate bone substitute materials in orthopedic surgery (5). These much larger HAP particles may play a different role in bone-rehabilitation in bone-defect areas. It has been reported that m-HAP particles effectively facilitate bone remodeling induced by osteoclasts and osteoblasts (5). Osteoclasts may be attracted to and mature on the surfaces of m-HAP particles *in vivo*, leading to the partial dissolution of m-HAP particles. Simultaneously, nearby osteoblasts are activated and new bone formation follows (26). In the cell culture condition using osteoblasts, bone-remodeling conditions are lacking. Large m-HAP particles cannot be digested by osteoblastic SaOS-2 cells. The more hydrophobic and less protein absorbed surfaces of m-HAP particles in the m-HAP/Col composite (27) may detach the SaOS-2 cells with time, rendering strange morphological changes, as observed in our study (Fig. 7). As a result, the osteogenic differentiation of SaOS-2 cells cultured on m-HAP/Col specimens may be sluggish, compared with those cultured on n-HAP/Col.

Col itself does not have strong osteoconductive properties (28). In brief, we prepared Col, n-HAP/Col and m-HAP/Col specimens, and conducted an expression analyses of four osteogenic differentiation marker genes in SaOS-2 cells cultured for 1, 2, 3 and 4 weeks on three test materials (Col, n-HAP/Col and m-HAP/Col) by RT-PCR. We then observed morphological changes under an SEM. The following experimental results were obtained: i) the osteogenic differentiation of SaOS-2 osteoblastic cells cultured on three-dimensional Col, n-HAP/Col and m-HAP/Col was considerably superior to that of cells cultured on two-dimensional polystyrene dishes (control); ii) the osteogenic differentiation of SaOS-2 cells cultured on n-HAP/Col was the most significant, followed by m-HAP/Col, whilst the differentiation of cells cultured on Col was the most insignificant; iii) the SaOS-2 cells phagocytized the n-HAP particles in the n-HAP/Col composite, leading to the acceleration of osteogenic differentiation; iv) the SaOS-2 cells had limited contact with the m-HAP particles in the m-HAP/Col composite, inducing inferior osteogenic differentiation.

## Figures and Tables

**Table I:** Primers of the five genes used for RT-PCR.

Genes	Sequences
β-actin	F: TGGCACCCAGCACAATGAA R: CTAAGTCATAGTCCGCCTAGAAGCA
ALP	F: GGACCATTCCCACGTCTTCAC R: CCTTGTAGCCAGGCCCATTG
COL1	F: TCTAGACATGTTCAGCTTTGTGGAC R: TCTGTACGCAGGTGATTGGTG
BSP	F: GGCCACGATTTATCTTTACAAGCA R: TCACCCTCAGAGTCTTCATCTTCA
BGLAP	F: AGGTGCAGCCTTTGTGTCCA R: GGCTCCCAGCCATTGATACAG

ALP, alkaline phosphatase; COL1, type 1 collagen; BSP, bone sialoprotein; BGLAP, bone gamma-carboxyglutamate (gla) protein (osteocalcin precursor).
